# Locomotor performance of cane toads differs between native-range and invasive populations

**DOI:** 10.1098/rsos.170517

**Published:** 2017-07-12

**Authors:** Georgia Kosmala, Keith Christian, Gregory Brown, Richard Shine

**Affiliations:** 1 School of Life and Environmental Sciences, University of Sydney, Sydney, New South Wales 2006, Australia; 2 Research Institute for the Environment and Livelihoods, Charles Darwin University, Casuarina, Northern Territory 0909, Australia

**Keywords:** introduced species, *Bufo marinus*, dehydration, temperature, locomotion

## Abstract

Invasive species provide a robust opportunity to evaluate how animals deal with novel environmental challenges. Shifts in locomotor performance—and thus the ability to disperse—(and especially, the degree to which it is constrained by thermal and hydric extremes) are of special importance, because they might affect the rate that an invader can spread. We studied cane toads (*Rhinella marina*) across a broad geographical range: two populations within the species' native range in Brazil, two invasive populations on the island of Hawai'i and eight invasive populations encompassing the eastern, western and southern limits of the toad invasion in Australia. A toad's locomotor performance on a circular raceway was strongly affected by both its temperature and its hydration state, but the nature and magnitude of those constraints differed across populations. In their native range, cane toads exhibited relatively low performance (even under optimal test conditions) and a rapid decrease in performance at lower temperatures and hydration levels. At the other extreme, performance was high in toads from southern Australia, and virtually unaffected by desiccation. Hawai'ian toads broadly resembled their Brazilian conspecifics, plausibly reflecting similar climatic conditions. The invasion of Australia has been accompanied by a dramatic enhancement in the toads' locomotor abilities, and (in some populations) by an ability to maintain locomotor performance even when the animal is cold and/or dehydrated. The geographical divergences in performance among cane toad populations graphically attest to the adaptability of invasive species in the face of novel abiotic challenges.

## 1. Introduction

Environmental conditions drive much of the variation in organismal performance, at a variety of ecological and evolutionary timescales. At one extreme, abiotic factors directly constrain the performance of individuals [1–9]; and at the other extreme, thermal and hydric conditions can act as selective forces on performance capabilities of a population and, ultimately, a species [10–14]. At intermediate timescales, physiological acclimation can adjust the immediate impact of abiotic variation on performance[15–20]. While acclimation of individuals usually occurs only over the range of conditions likely to be encountered by a population, sustained exposure to novel environmental challenges can favour evolutionary changes that extend the range of conditions over which an organism can operate successfully [10–12,14].

Many environments worldwide are experiencing rapid changes in thermal and hydric conditions, and such changes look set to continue at an increasing pace [21]. Will existing adaptations to ancestral climates prevent organisms from functioning effectively in shifting abiotic conditions? Invasive species provide an ideal opportunity to answer that question for two reasons. The first reason is that introduced populations are subjected to environmental conditions very different from those experienced in the native range. That difference allows us to investigate the rate at which a novel selective pressure or direct environmental influence can modify ancestral performance traits. In performance traits that are simultaneously affected by multiple abiotic factors (e.g. temperature and moisture), comparisons between native-range and invasive populations also can clarify the degree to which correlated responses to multiple constraints can be teased apart by plasticity or evolution. The short and well-documented timeframes of many invasions, coupled with a capacity for behavioural and physiological flexibility and rapid evolutionary change in many introduced taxa [22–26], allow us to compare closely related organisms that are exposed to very different challenges [27,28]. Second, locomotor performance is a central determinant of dispersal rate, a trait that can evolve very rapidly within invasive populations [29,30]. How quickly an animal moves through its environment, and how well it can withstand climatic extremes while doing so, can directly influence an invasive species' dispersal rate and thus, its distribution. For an invader moving into a novel climate, a high level of performance under ancestral conditions is not enough; it must also be able to perform in the newly encountered conditions [31,32].

Locomotor ability of anurans is well suited to analyses of these questions, because an anuran's ability to disperse may depend upon both its temperature and its hydration state [6,7,31,33–37]. To understand how local environments constrain locomotor performance in such an animal, we thus need to take our measurements over a range of abiotic conditions [36]. To understand how adaptation and/or developmental plasticity have modified those norms of reaction, we can compare animals that have been subjected to different selective pressures and environmental experiences [36].

Cane toads (*Rhinella marina*; formerly *Bufo marinus*) are large bufonid anurans native to relatively hot, wet areas of Central and South America [38]. In the early part of the twentieth century, the species was introduced to more than 40 countries around the world, in a misguided attempt at biological control of insect pests [22–26,38]. Some of those recipient countries have climates similar to those within the toads' native range, but others pose novel thermal and hydric challenges. Notably, the toads' invasion across tropical Australia has exposed it to climates much drier and hotter than occur within its native range [27,28,39–42]. Given that anuran locomotor performance is constrained both by temperature and by moisture [27,28], how has the toads' invasion of novel climatic zones affected the sensitivity of its locomotor ability to thermal and hydric conditions? Specifically, has the cane toad's invasion of Australian areas with harsh climates been accompanied by an enhanced tolerance of hot and desiccating conditions?

## 2. Material and methods

### 2.1. Study species

Cane toads (*R. marina*) are large (exceptionally, to greater than 1 kg) ‘true toads’ (family Bufonidae) [26]. The species' native range extends from Mexico and southern Texas through an extensive area of Central and South America [22,26,38]. Adult cane toads are heavyset terrestrial anurans that feed on a diversity of invertebrate prey [26,38]. The toads' large body sizes and prodigious appetites encouraged commercial sugarcane growers to import toads to control insect pests in plantations in Puerto Rico; and from there, 150 toads were translocated to Hawai'i in 1932 and released in sugarcane fields [22,43]. Toads are still common on the major Hawai'ian islands, where the animals are relatively sedentary [44].

The 101 descendants of the Hawai'ian immigrants were collected in 1935 and shipped to northeastern Australia, where thousands of their progeny were released along the Queensland (QLD) coast [22]. The anurans have since spread widely through tropical and subtropical regions of Australia, inflicting major impacts on populations of anuran-eating predators [23–25,45]. In Australia, cane toads thrive not only in well-watered regions of coastal northeastern Australia, but also in severely arid regions [28,46]. In recent years, toads have also invaded cold montane regions of southeastern Australia [20,41]. Dispersal rates are dramatically higher for individuals at the (western) invasion front than in individuals from range-core areas of eastern Australia [29,47]. Analyses of climatic correlates of cane toad distributions led Tingley *et al*. [48] to conclude that cane toads occupy a wider range of climatic conditions in Australia than in their native range.

### 2.2. Sampling locations

We collected adult toads (both males and females, ranging from 50 to 300 g) from locations in their native range (Brazil), in Hawai'i and in Australia. All toads were collected by hand at night, placed in damp cloth bags and kept in a moist, cool environment to reduce stress. Following capture, toads were transported to local laboratory facilities for the experiments. All toads collected were tested, and sample numbers are detailed in table 1.

**Table 1.** Details of toad sampling sites and the annual climatic conditions of each location. Climatic data sourced from Climate-Data.org [49]. PA, Pará; AM, Amazonas; HI, Hawai'i; WA, Western Australia; NT, Northern Territory; QLD, Queensland; NSW, New South Wales. Am, equatorial monsoonal; Af, equatorial fully humid; Aw, equatorial winter dry; BSh, arid steppe hot arid; Cfa, warm temperate fully humid hot summer.

country	location	year of introduction	latitude/longitude	mean annual rainfall^a^	mean annual temperature^a^	climate classification^b^	# ♀	# ♂	acclimation
Brazil	Alter do Chão (PA)	—	2°30'5.45′′ S/	1991 mm	25.9°C	Am	0	20	4
			54°57'25.76′′ W	(34–346 mm)	(25.1–26.9°C)				
	Manaus (AM)	—	3° 0'32.04′′ S/	2145 mm	27.4°C	Am	9	11	9
			59°56'49.84′′ W	(56–295 mm)	(26.9–28.2°C)				
USA	Hilo (HI)	1932	19°41'58.53′′ N/	3459 mm	23.1°C	Af	8	12	14
			155° 4'51.79′′ W	(177–397 mm)	(21.7–24.6°C)				
	Kailua- Kona (HI)	1932	19°49'12.38′′ N/	862 mm	23.5°C	Aw	8	12	7
			155°50'11.04′′ W	(55–88 mm)	(22.0–24.9°C)				
Australia	Kununurra (WA)	2011	15°46'27.14′′ S/	720 mm	28.8°C	BSh	13	7	7
			128°44'24.51′′ E	(0–186 mm)	(23.3–32.6°C)				
	Oombulgurri (WA)	2013	15°10'49.35′′ S/	718 mm	29.4°C	BSh	18	2	7
			127°50'42.55′′ E	(0–181 mm)	(24.3–32.9°C)				
	Leaning Tree Lagoon (NT)	2006	12°42'26.42′′ S/	1500 mm	27.2°C	Aw	10	10	4
			131°25'12.80′′ E	(1–364 mm)	(23.9–29.4°C)				
	Katherine (NT)	2010	14°27'48.81′′ S/	1009 mm	27.5°C	Aw	12	7	7
			132°15'36.38′′ E	(0–250 mm)	(22.1–31.6°C)				
	Charters Towers (QLD)	1953	20°4'34.56′′ S/	692 mm	23.2°C	BSh	13	7	7
			146°15'30.67′′ E	(8–142 mm)	(17.3–27.4°C)				
	Townsville (QLD)	1935	19°15'27.44′′ S/	1111 mm	24.1°C	Aw	13	7	7
			146°49'4.36′′ E	(9–275 mm)	(19.0–27.6°C)				
	Brooms Head (NSW)	2005	29°36'22.84′′ S/	1471 mm	19.2°C	Cfa	8	4	7
			153°20'8.97′′ E	(49–188 mm)	(13.8–23.6°C)				
	Tabbimoble (NSW)	2010	29°11'58.59′′ S/	1558 mm	19.4°C	Cfa	8	4	7
			153°16'13.47′′ E	(52–193 mm)	(14.0–23.6°C)				

^a^Values in parentheses indicate the range of the mean monthly values for rainfall and temperature.

^b^Köppen and Geiger Climate Classification System. Am, equatorial monsoonal; Af, equatorial fully humid; Aw, equatorial winter dry; BSh, arid steppe hot arid.

Toads from the native range were collected in Manaus, Amazonas (AM) and Alter do Chão, Pará (PA) in Brazil. Fieldwork occurred during January and February 2015, a warm and wet time of year.

During June and July 2015, we collected toads on the island of Hawai'i (HI, USA), from sites in the extreme east (Hilo) and extreme west (Kailua-Kona) of the island. The eastern (windward) side is humid and warm, whereas the western (leeward) side is much drier, due to a rainshadow effect coupled with highly porous volcanic soils [44]. Cane toads are broadly distributed through the landscape on the (wetter) eastern side of the island, but largely restricted to anthropogenically watered sites (golf courses) on the drier western side [44].

In Australia, we collected toads from eight sites. Two sites in Western Australia (WA) (Oombulgurri, Kununurra) were in the extreme west of the species' range, close to the invasion front (less than 2 years post-colonization). The climate is hot year-round and seasonally arid. Another two sites were in the Northern Territory (NT) (Katherine, Leaning Tree Lagoon) in an area exposed to a similar but less harsh climate than that encountered further to the west. Our two QLD sites (Townsville, Charters Towers) experience moister conditions year-round. Lastly, our two sites in New South Wales (NSW) (Brooms Head and Tabbimoble) are close to the current southeastern invasion front, and experience cooler (but generally moist) conditions (table 1 for details of site locations, invasion history, climatic conditions and sample sizes). Climate data were sourced from Climate-Data.org [49].

### 2.3. Husbandry and experimental methods

After capture, toads were allowed to acclimate in laboratory conditions for at least two weeks. During acclimation (and between trials), we fed the anurans crickets and mealworms, and provided ad libitum access to water and shelter. The room was set to 25°C, with a 12 L : 12 D cycle. Prior to their first locomotor trials, we maintained the toads at the test temperature for a minimum of 2 h, during which time we kept them in water to ensure full hydration. We emptied the toads' bladders by gently applying pressure to the abdomen, and then encouraged them to run along a circular wooden track inside a temperature-controlled room, and stimulated them to keep moving by gentle pokes to their urostyles if needed. The trial continued for 10 min. We recorded the number of laps, plus additional distance on the uncompleted final lap for each individual. Because all trials were the same duration, we used total distance travelled as our index of locomotor performance. To correct for size variation among toads (snout–vent lengths (SVL) ranged from 85.6 to 183.9 mm), we expressed distances in terms of body lengths travelled during a trial as the dependent variable in our statistical analyses—we will refer to this variable as ‘performance’.

After the initial trials, we placed toads in desiccating conditions (exposed to a flow of dry air), and allowed them to dehydrate overnight until they lost 10% of their initial (fully hydrated, empty bladder) body mass. Toads were not placed in a chamber, but rather stayed overnight in mesh containers, inside the temperature-controlled room at 25°C and with airflow provided by the room's air-conditioning system and fans. This allowed for a slower, gentler, more natural water loss than in wind tunnels/chambers. All further desiccation processes occurred in this same manner, regardless of the temperature being tested during the locomotor performance. We repeated the locomotor test protocol over the following days to measure locomotor performance of toads at 90%, 80% and 70% of their body mass. After the final trials, we placed toads back into water (at room temperature) to allow full recovery. Each toad was tested at 15, 25 and 35°C (except for Hawai'ian toads, in which case only 25 and 35°C trials were performed because we were unable to maintain low temperatures in our test facilities in Hawai'i). We tested all animals at 25°C first, because it was the temperature of acclimation and close to the natural environmental temperature (i.e. it allowed us to establish a baseline performance). Following that, we tested each animal at 15°C, and lastly at 35°C.

### 2.4. Statistical analyses

Using the open-access software R Studio v. 0.99.893 [50], we used linear mixed models (package lmer) to evaluate the fixed effects of test conditions (temperature and hydration level) and location on the toads' locomotor performance. We included individual ID no. as a random factor in the analyses to accommodate multiple measures taken from individual toads. Collection site and country were combined as groups (per state for Australian toads, and per country for Hawai'ian and Brazilian toads). We treated test temperature and desiccation level as three- and four-level categorical variables, respectively.

We ran three sets of analyses. First, we combined data from all toads to quantify overall patterns of the influence of the two categorical variables (test temperature and dehydration level) on locomotor performance. Next, we included country of origin (Brazil, USA, Australia) as an additional fixed factor, to compare locomotor responses with environmental conditions at this broad level. Lastly, we restricted the dataset to Australian toads only, and included state of origin (QLD, NSW, NT, WA) as a fixed effect, to look in more detail at the divergence in locomotor traits over the 80-year history of toad invasion in this continent. Residuals from all analyses conformed to assumptions of normality and homoscedastic variances. The data used in these analyses were deposited at Dryad Digital Repository [51].

## 3. Results

### 3.1. Overall effects of temperature and hydration on locomotor performance of toads

**Figure 1.**
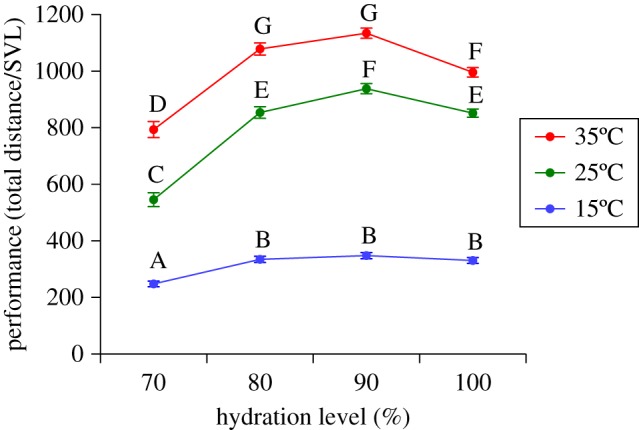
Effect of temperature and hydration level treatments on overall performance. Performance was expressed as the total distance travelled divided by the individual's SVL. Letters above symbols represent groupings from the Tukey *post hoc* tests. Symbols with the same letter are not significantly different from one another. The graph shows mean values (±s.e.m.), based on *N* = 2172 data points, collected from 209 animals, pooled from all populations.

We combined data from all populations to explore overall impacts of temperature and hydration on locomotor performance. Toads exhibited wide variation in locomotor ability, as a function of test conditions as well as traits of toads (total distance travelled ranged from 30 to 18 560 cm over the 10-min test). ANOVA on the combined data showed that a toad's locomotor performance was affected by a significant interaction between its temperature and its hydration level (*F*_6,1955_ = 30.39, *p* < 0.001; figure 1; see also electronic supplementary material, table S9). Thus, the effect of hydration on locomotion depended on the temperature. At all tested temperatures, there was a steep decrease in locomotion when toads were dehydrated below 80%. However, in the lowest test temperature (15°C), locomotor performance was significantly better at 80% hydration and remained stable at higher levels (90%, 100%), while at higher test temperatures (25°C, 35°C), performance was significantly lower at 100% hydration than at 80 and 90% (based on general patterns in figure 1; see also electronic supplementary material, table S1). Furthermore, toads travelled farther under warmer conditions, and the significant interaction term reflects a trend for locomotor performance to be low, regardless of hydration level, at the lowest test temperature (figure 1).

### 3.2. Comparison of toads from Brazil versus Hawai'i versus Australia

**Figure 2.**
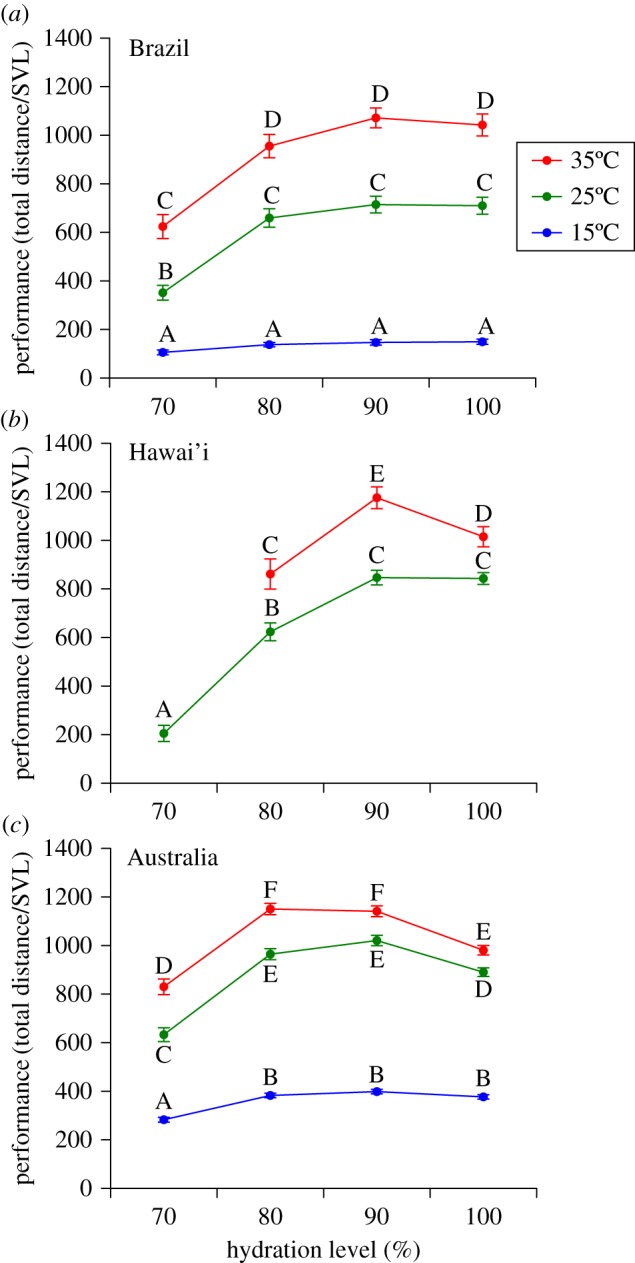
Comparisons between the locomotor performance of cane toads from the native range and two areas to which they have been introduced. The panels represent the average performance of toads from (*a*) Brazil, (*b*) Hawai'i and (*c*) Australia at each of the test treatments (temperature + hydration level). Letters above symbols represent groupings from the Tukey *post hoc* tests. Within each panel (but not among panels), symbols with the same letter are not significantly different from one another. Hawai'ian toads could not be tested at 15°C or at the 70% hydration level at 35°C. Graphs show mean values (±s.e.m.) based on *N* = 2172 data points, collected from Brazil = 35, Hawai'i = 38 and Australia = 136 animals.

We explored the effects of temperature, hydration level and country of origin in a linear mixed-effects ANOVA model, including individual identity as a random effect. The model resulted in a significant three-way interaction term (*F*_8,1938_ = 4.50, *p* < 0.001; see also electronic supplementary material, table S10), indicating that the effect of temperature on locomotor performance depended on hydration status (as above) but also that the nature of this dependency varied among locations (figure 2). A *post hoc* test comparing means clarifies this interaction (figure 2; electronic supplementary material, tables S2–S4). Most notably, Australian populations maintained locomotor performance under a wider range of conditions than did Brazilian or Hawai'ian toads.

Decreasing the hydration level from 100 to 90% had little impact on the locomotor performance of Brazilian toads at either 25 or 35°C, and this decrease in hydration level slightly enhanced performance of Australian toads. By contrast, decreasing hydration level from 90 to 80% dramatically reduced locomotor performance of Hawai'ian toads at both 25 and 35°C. This decrease was less marked in Brazilian toads, and minimal in Australian toads (electronic supplementary material, tables S2–S4).

When tested at low temperature, Australian toads travelled almost twice as far as did either Brazilian or Hawai'ian toads. Brazilian and Hawai'ian toads were greatly affected by dehydration, especially at high temperatures. At 70% hydration and 35°C, Hawai'ian toads were rendered immobile, which necessitated excluding them from this treatment combination (figure 2).

### 3.3. Comparison among toads from different locations in Australia

**Figure 3.**
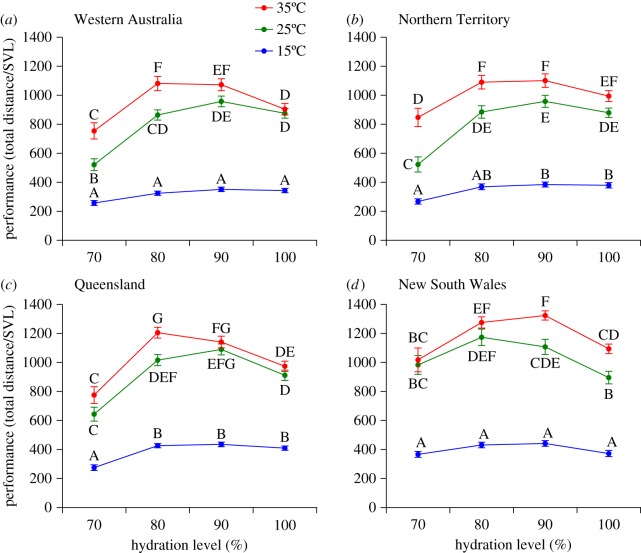
Comparison of the locomotor performance of cane toads from different populations in Australia. The panels represent the average performance of toads from (*a*) WA, (*b*) NT, (*c*) QLD and (*d*) NSW at each of the test treatments (temperature + hydration level). Letters above symbols represent groupings from the Tukey *post hoc* tests. Within each panel (but not among panels), symbols with the same letter are not significantly different from one another. Graphs show mean values (±s.e.m.), based on *N* = 1579 data points, collected from WA = 35, NT = 39, QLD = 40 and NSW = 22 animals.

Among Australian toads, a significant three-way interaction (*F*_18,1399_ = 2.67, *p* < 0.001; see also electronic supplementary material, table S11) indicated that the manner in which temperature and hydration level affected locomotor performance depended on which population the toad was from. Comparing among Australian populations, toads from NSW had much higher locomotor performance than did toads from any of the other populations. The NSW animals not only had higher performance at 90 and 80% hydration level at all tested temperatures, but also were more resilient to the 70% hydration treatment (i.e. exhibited less decrease in performance: figure 3). Toads from all Australian regions performed best at 35°C, at all hydration levels (figure 3; electronic supplementary material, tables S5–S8).

Following the models analysed above, we performed pairwise comparisons of significant factors and interactions using the Tukey method, within each population, to elucidate patterns of effect. Both test temperature and hydration level significantly affected toad performance, but in different ways in different populations (electronic supplementary material, tables S5–S8).

## 4. Discussion

Test conditions (temperature and hydration level) strongly affected the locomotor performance of our cane toads, consistent with previous studies on other anuran species, including bufonids [17,28,31,34,36,52,53]. Although we encouraged them to continue moving, toads travelled shorter distances when they were cool and/or dehydrated. Importantly, performance of these animals was also affected by an interaction between these two factors, such that the effect of desiccation on distance travelled differed among test temperatures. Thermal impacts on performance were relatively straightforward in all tested populations (both native and introduced), with toads moving further at higher temperatures. However, hydric condition (level of desiccation) had more complex effects. At warmer temperatures, toads performed optimally at intermediate levels of hydration, and travelled shorter distances at hydration levels above and below this optimum (figure 1). At warmer temperatures, Australian and Hawai'ian toads unexpectedly exhibited a decrease in locomotor performance when hydration level increased from 90 to 100%. Full hydration may impose a physiological constraint, or alter behavioural factors that make toads less inclined to perform to the same level in experimental trials [54].

Only a few studies have looked into the combined effects of temperature and hydration levels in *R. marina*. Tingley *et al*. [28] compared the effect of different hydration levels in two populations of toads (a mesic and a semi-arid population). However, they only tested locomotion at approximately 25°C, with no 70% hydration treatment; also, their toads were dehydrated rapidly in a wind tunnel, whereas in our study toads were dehydrated overnight. Slower dehydration better mimics the natural situation, and may elicit more natural behavioural and physiological responses. Tingley *et al*. [28] suggested that locomotor performance of toads from a mesic area (corresponding to our QLD populations) declined more rapidly with dehydration than did that of toads from a semi-arid area (corresponding to our NT populations). Our study confirms this pattern, but including the WA and NSW populations reveals a greater complexity: locomotion of toads from the western invasion front (WA) is more sensitive to both temperature and dehydration than is that of toads from the southeastern invasion front (NSW). Although warmer temperatures were associated with better performance overall, the generally higher performance levels of toads from NSW suggest that lower temperatures (or greater thermal variance) pose more of a challenge to *R. marina* than does dehydration.

Tingley *et al*. [48] concluded that the southern limits of the cane toad's native range in Brazil are driven by competition with a closely related species (*Rhinella schneideri*), rather than abiotic constraints. However, our results suggest that cane toads perform poorly in cool dry environments, suggesting that abiotic factors may be important also. Australian toads performed better than native-range conspecifics under all tested conditions, suggesting that individuals in these invasive populations are behaviourally and/or physiologically more capable of tolerating extreme conditions.

Consistent with the prediction that toads will exhibit enhanced locomotor performance as they invade harsher environments, our comparisons among toads from different parts of Australia also reveal finer-scale geographical divergences in the sensitivity of locomotor performance to test conditions (figure 3). For example, toads from the southeastern invasion front (NSW) continued to move along the raceway even when cool and highly dehydrated, whereas toads from tropical populations (QLD and NT) were more affected by dehydration and cold. The magnitude of such differences was striking: for example, toads from NSW travelled an average of 39.08 ± 9.62 m at 15°C and 70% hydration, whereas toads from QLD travelled an average of 29.01 ± 12.13 m under the same conditions; Brazilian toads moved an average of 11.57 ± 5.68 m under those conditions, and we were unable to even test Hawai'ian animals under those conditions because they were immobile and unresponsive.

The substantial divergences among populations might be due to phenotypic plasticity (responses to environmental conditions experienced during the animal's lifetime) and/or to adaptation that has occurred during the relatively brief period since the groups were separated. Toads were brought from the South American mainland to Puerto Rico in 1923, and also perhaps earlier [43]. Puerto Rican toads were then translocated to Hawai'i in 1932, and from there to QLD in 1935 [43]. Rather than evolving such strong divergences in locomotor performance over this short period, the divergences may have been driven by phenotypic plasticity rather than evolutionary adaptation. Because we worked with field-collected toads, their responses to temperature and desiccation may have been fashioned by the individual's own experiences prior to collection. Toads from different regions within Australia differ strongly in a suite of phenotypic traits related to dispersal rate (encompassing morphology, physiology and behaviour), and common-garden studies have shown that many of those geographical differences are heritable [29,55,56]. Nonetheless, we have no evidence that geographical shifts in thermal and hydric sensitivity of performance also have a genetic underpinning.

What mechanisms may underlie the development of a phenotype capable of continuing to move around even in cool and dry conditions? The obvious explanation is geographical divergence in climates. Cane toads in their native range, and in Hawai'i, are exposed primarily to relatively stable warm moist conditions, through a combination of prevailing climates and active selection of moist habitats [44]. Resistance to cool dry environmental conditions is unlikely to have been an important determinant of fitness. By contrast, Australian populations of the cane toad have been exposed to novel (and often, harsh) abiotic conditions since their arrival in that continent 80 years ago [48]. An ability to continue dispersing even under such conditions may have conferred a significant fitness benefit in Australia, leading to the evolution (or phenotypically plastic manifestation) of a phenotype capable of maintaining locomotor performance even under abiotic extremes. In keeping with that interpretation, the Australian populations most resistant to cool conditions are those from NSW, the area where toads are most likely to encounter cool weather [20]. The sites where we collected toads also vary in features such as distances between potential spawning-ponds, and the array of local predators; such factors also may influence selection on locomotor ability. To know whether such ability is innate or phenotypically plastic (expressed only when such conditions are encountered during an animal's lifetime) would require studies on captive-raised animals.

The success of introduced species often depends on their ability to deal with challenges imposed by the abiotic environment, rather than local biotic resistance [57]. As a result, populations of invasive species can diverge in physiological responses as a result of being subject to different conditions in different areas [58,59]. Our data on cane toads accord well with those conclusions, and suggest that rapid adjustments to deal with novel abiotic challenges may allow cane toads to extend into larger areas of Australia than would be expected from their ‘climatic envelope’ within the native range, or within already-invaded parts of Australia. The implications for wildlife managers are clear: we may need effective means to control cane toads not only in the kinds of environments in which they currently occur, but also in other (cooler, drier) regions currently well outside the predicted final extent of cane toad distributions in Australia.

In summary, our data add to a growing picture of cane toads as extraordinarily flexible animals. The species evolved in relatively benign environments [48], favouring an ancestral condition of low resistance to thermal and hydric extremes. That sensitivity, if retained, would have precluded successful colonization by cane toads in many of the places to which it was translocated [26]. Instead, the cane toad changed rapidly after it encountered novel abiotic conditions in its introduced range. Despite the low numbers of founding individuals in successive bottlenecks in Hawai'i and QLD, and thus low genetic diversity [60], toads rapidly evolved distinctive modifications in a diverse suite of phenotypic traits [29,55,56], and managed to thrive and spread over large portions of the driest continent on Earth. This study suggests that the toads' success in Australia is also due at least partly to their ability to maintain effective locomotion even under hot and desiccating conditions. The toads' success in Australia is discouraging, because cane toads have had severe ecological impacts in that continent [45], and at the same time encouraging, because it testifies to the ability of organisms to deal with the kinds of novel environmental challenges that are increasingly being thrust upon them. And it reinforces a cautionary note from other studies: we cannot reliably infer the attributes of an invasive species from studies in the organism's native range, because the process of invasion may generate rapid divergence.

## Supplementary Material

Supporting Information

## Data accessibility

Data are available at the external repository Dryad: http://dx.doi.org/10.5061/dryad.d9n5d [51].
