# Supplementary material for: Locomotor performance of cane toads differs between native-range and invasive populations
Source: R Soc Open Sci. 2017 Jul 12;4(7):170517. doi: 10.1098/rsos.170517 (PMC5541569; doi:10.1098/rsos.170517)
Supplement: Supporting Information [file rsos170517supp1.doc]

**Supporting Information**

Results of Tukey post-hoc analyses comparing locomotor performance of toads from populations in Brazil, Hawaii and Australia.

**Table S1. Pairwise comparisons using Tukey post-hoc analyses on the effects of temperature and hydration on all tested toads (overall effect)**

| Temperature (^o^C) | Hydration level (%) | Least Mean Squares | SE | df | Group |
| --- | --- | --- | --- | --- | --- |
| 15 | 70 | 219.48 | 18.34 | 743.21 | A |
| 15 | 80 | 306.87 | 18.31 | 738.99 | B |
| 15 | 100 | 309.67 | 18.18 | 722.36 | B |
| 15 | 90 | 321.31 | 18.28 | 734.78 | B |
| 25 | 70 | 539.67 | 17.70 | 662.95 | C |
| 35 | 70 | 758.26 | 18.55 | 769.36 | D |
| 25 | 100 | 851.42 | 17.03 | 585.71 | E |
| 25 | 80 | 851.94 | 17.08 | 591.04 | E |
| 25 | 90 | 937.67 | 17.03 | 585.71 | F |
| 35 | 100 | 974.05 | 17.68 | 660.01 | F |
| 35 | 80 | 1056.68 | 17.68 | 660.01 | G |
| 35 | 90 | 1112.42 | 17.68 | 660.01 | G |

**Table S2. Pairwise comparisons using Tukey post-hoc analyses on the effects of temperature and hydration on toads from Brazil (native range population)**

| Temperature (^o^C) | Hydration level (%) | Least Mean Squares | SE | df | Group |
| --- | --- | --- | --- | --- | --- |
| 15 | 70 | 105.86 | 33.14 | 167.89 | A |
| 15 | 80 | 137.84 | 33.14 | 167.89 | A |
| 15 | 90 | 146.91 | 32.78 | 162.99 | A |
| 15 | 100 | 152.71 | 32.42 | 158.36 | A |
| 25 | 70 | 354.64 | 32.42 | 158.36 | B |
| 35 | 70 | 620.13 | 34.75 | 190.24 | C |
| 25 | 80 | 659.11 | 32.06 | 154.11 | C |
| 25 | 100 | 709.70 | 32.06 | 154.11 | C |
| 25 | 90 | 714.42 | 32.06 | 154.11 | C |
| 35 | 80 | 949.46 | 33.52 | 173.06 | D |
| 35 | 100 | 1036.42 | 33.52 | 173.06 | D |
| 35 | 90 | 1065.69 | 33.52 | 173.06 | D |

**Table S3. Pairwise comparisons using Tukey post-hoc analyses on the effects of temperature and hydration on toads from Hawai’i (older introduction)**

| Temperature (^o^C) | Hydration level (%) | Least Mean Squares | SE | df | Group |
| --- | --- | --- | --- | --- | --- |
| 25 | 70 | 255.26 | 48.35 | 178.48 | A |
| 25 | 80 | 616.74 | 35.03 | 105.08 | B |
| 35 | 80 | 821.16 | 39.89 | 138.57 | C |
| 25 | 100 | 843.07 | 34.34 | 100.51 | C |
| 25 | 90 | 846.68 | 34.34 | 100.51 | C |
| 35 | 100 | 974.85 | 39.89 | 138.57 | D |
| 35 | 90 | 1135.32 | 39.89 | 138.57 | E |
| 35 | 70 | NA | NA | NA | NA |

**Table S4. Pairwise comparisons using Tukey post-hoc analyses on the effects of temperature and hydration on toads from Australia (younger introduction)**

| Temperature (^o^C) | Hydration level (%) | Least Mean Squares | SE | df | Group |
| --- | --- | --- | --- | --- | --- |
| 15 | 70 | 279.79 | 20.13 | 414.27 | A |
| 15 | 100 | 373.99 | 19.97 | 403.46 | B |
| 15 | 80 | 376.53 | 20.10 | 412.26 | B |
| 15 | 90 | 391.95 | 20.10 | 412.26 | B |
| 25 | 70 | 666.34 | 19.94 | 401.20 | C |
| 35 | 70 | 834.91 | 20.20 | 418.84 | D |
| 25 | 100 | 890.25 | 19.88 | 397.28 | D |
| 35 | 100 | 977.51 | 20.20 | 418.84 | E |
| 25 | 80 | 984.35 | 19.88 | 397.28 | E |
| 25 | 90 | 1027.92 | 19.88 | 397.28 | E |
| 35 | 90 | 1145.70 | 20.20 | 418.84 | F |
| 35 | 80 | 1149.22 | 20.20 | 418.84 | F |

**Table S5. Pairwise comparisons using Tukey post-hoc analyses on the effects of temperature and hydration on toads from Western Australia (western invasion front)**

| Temperature (^o^C) | Hydration level (%) | Least Mean Squares | SE | df | Group |
| --- | --- | --- | --- | --- | --- |
| 15 | 70 | 257.12 | 35.08 | 115.89 | A |
| 15 | 80 | 324.17 | 35.08 | 115.89 | A |
| 15 | 100 | 342.86 | 35.08 | 115.89 | A |
| 15 | 90 | 350.89 | 35.08 | 115.89 | A |
| 25 | 70 | 521.05 | 35.08 | 115.89 | B |
| 35 | 70 | 749.40 | 35.38 | 119.17 | C |
| 25 | 80 | 863.87 | 35.08 | 115.89 | CD |
| 25 | 100 | 875.41 | 35.08 | 115.89 | D |
| 35 | 100 | 899.03 | 35.38 | 119.17 | D |
| 25 | 90 | 957.35 | 35.08 | 115.89 | DE |
| 35 | 90 | 1067.84 | 35.38 | 119.17 | EF |
| 35 | 80 | 1076.56 | 35.38 | 119.17 | F |

**Table S6. Pairwise comparisons using Tukey post-hoc analyses on the effects of temperature and hydration on toads from the Northern Territory (range core)**

| Temperature (^o^C) | Hydration level (%) | Least Mean Squares | SE | df | Group |
| --- | --- | --- | --- | --- | --- |
| 15 | 70 | 264.86 | 38.49 | 94.49 | A |
| 15 | 80 | 368.77 | 38.26 | 92.48 | AB |
| 15 | 100 | 379.26 | 38.26 | 92.48 | B |
| 15 | 90 | 384.39 | 38.26 | 92.48 | B |
| 25 | 70 | 525.57 | 38.48 | 94.43 | C |
| 35 | 70 | 841.11 | 38.73 | 96.59 | D |
| 25 | 100 | 879.23 | 38.26 | 92.48 | DE |
| 25 | 80 | 884.59 | 38.26 | 92.48 | DE |
| 25 | 90 | 957.76 | 38.26 | 92.48 | E |
| 35 | 100 | 987.59 | 38.73 | 96.59 | EF |
| 35 | 80 | 1083.75 | 38.73 | 96.59 | F |
| 35 | 90 | 1094.56 | 38.73 | 96.59 | F |

**Table S7. Pairwise comparisons using Tukey post-hoc analyses on the effects of temperature and hydration on toads from Queensland (long-colonized)**

| Temperature (^o^C) | Hydration level (%) | Least Mean Squares | SE | df | Group |
| --- | --- | --- | --- | --- | --- |
| 15 | 70 | 262.64 | 36.42 | 197.45 | A |
| 15 | 100 | 403.24 | 36.06 | 192.38 | B |
| 15 | 80 | 414.22 | 36.42 | 197.45 | B |
| 15 | 90 | 423.02 | 36.42 | 197.45 | B |
| 25 | 70 | 637.25 | 35.35 | 182.99 | C |
| 35 | 70 | 763.10 | 36.42 | 197.45 | C |
| 25 | 100 | 911.20 | 35.01 | 178.71 | D |
| 35 | 100 | 961.44 | 36.42 | 197.45 | DE |
| 25 | 80 | 1015.68 | 35.01 | 178.71 | DEF |
| 25 | 90 | 1089.53 | 35.01 | 178.71 | EFG |
| 35 | 90 | 1128.57 | 36.42 | 197.45 | FG |
| 35 | 80 | 1192.87 | 36.42 | 197.45 | G |

**Table S8. Pairwise comparisons using Tukey post-hoc analyses on the effects of temperature and hydration on toads from New South Wales (southeastern invasion front)**

| Temperature (^o^C) | Hydration level (%) | Least Mean Squares | SE | df | Group |
| --- | --- | --- | --- | --- | --- |
| 15 | 70 | 334.14 | 50.79 | 45.31 | A |
| 15 | 100 | 371.74 | 50.30 | 43.75 | A |
| 15 | 80 | 398.70 | 50.79 | 45.31 | A |
| 15 | 90 | 409.25 | 50.79 | 45.31 | A |
| 25 | 100 | 895.17 | 50.30 | 43.75 | B |
| 25 | 70 | 982.69 | 50.30 | 43.75 | BC |
| 35 | 70 | 985.66 | 50.79 | 45.31 | BC |
| 35 | 100 | 1061.57 | 50.79 | 45.31 | CD |
| 25 | 90 | 1107.02 | 50.30 | 43.75 | CDE |
| 25 | 80 | 1173.29 | 50.30 | 43.75 | DEF |
| 35 | 80 | 1243.32 | 50.79 | 45.31 | EF |
| 35 | 90 | 1291.44 | 50.79 | 45.31 | F |

**Table S9. ANOVA table for the linear mixed-effects model on the overall effects of temperature and hydration in all tested toads.**

|  | **Sum Sq** | **Mean Sq** | **NumDF** | **DenDF** | **F.value** | **Pr(>F)** |
| --- | --- | --- | --- | --- | --- | --- |
| Temperature | 165547918 | 82773959 | 2 | 1979.2 | 2892.43 | < 2.2e-16 *** |
| Hydration | 23677479 | 7892493 | 3 | 1956.2 | 275.79 | < 2.2e-16 *** |
| Temperature:Hydration | 5218017 | 869670 | 6 | 1954.8 | 30.39 | < 2.2e-16 *** |

**Table S10. ANOVA table for the linear mixed-effects model on the effects of temperature, hydration and country of origin in all tested toads.**

|  | Sum Sq | Mean Sq | NumDF | DenDF | F.value | Pr(>F) |
| --- | --- | --- | --- | --- | --- | --- |
| Temperature | 111072023 | 55536012 | 2 | 1972.48 | 2153.04 | < 2.2e-16 *** |
| Hydration | 13682393 | 4560798 | 3 | 1951.48 | 176.81 | < 2.2e-16 *** |
| Country | 1625043 | 812521 | 2 | 229.95 | 31.50 | 8.113e-13 *** |
| Temperature:Hydration | 3761425 | 626904 | 6 | 1937.50 | 24.30 | < 2.2e-16 *** |
| Temperature:Country | 1567365 | 522455 | 3 | 1977.51 | 20.25 | 6.284e-13 *** |
| Hydration:Country | 3091590 | 515265 | 6 | 1947.65 | 19.98 | < 2.2e-16 *** |
| Temperature:Hydration:Country | 928787 | 116098 | 8 | 1937.36 | 4.50 | 1.966e-05 *** |

**Table S11. ANOVA table for the linear mixed-effects model on the effects of temperature, hydration and population of origin in all Australian toads.**

|  | Sum Sq | Mean Sq | NumDF | DenDF | F.value | Pr(>F) |
| --- | --- | --- | --- | --- | --- | --- |
| Temperature | 124284449 | 62142225 | 2 | 1404.15 | 2537.74 | < 2.2e-16 *** |
| Hydration | 15886868 | 5295623 | 3 | 1399.34 | 216.26 | < 2.2e-16 *** |
| Population | 355876 | 118625 | 3 | 131.86 | 4.84 | 0.0031337 ** |
| Temperature:Hydration | 3202283 | 533714 | 6 | 1399.34 | 21.80 | < 2.2e-16 *** |
| Temperature:Population | 1450445 | 241741 | 6 | 1404.31 | 9.87 | 1.094e-10 *** |
| Hydration:Population | 1393680 | 154853 | 9 | 1399.33 | 6.32 | 8.107e-09 *** |
| Temperature:Hydration:Population | 1176572 | 65365 | 18 | 1399.34 | 2.67 | 0.0001779 *** |
